# DNA methylation‐mediated silencing of microRNA‐204 enhances T cell acute lymphoblastic leukemia by up‐regulating MMP‐2 and MMP‐9 via NF‐κB

**DOI:** 10.1111/jcmm.15896

**Published:** 2021-02-10

**Authors:** Congmeng Lin, Dabing Chen, Tingting Xiao, Dandan Lin, Dayi Lin, Luhui Lin, Haojie Zhu, Jingjing Xu, Wenwen Huang, Ting Yang

**Affiliations:** ^1^ Department of Hematology Zhangzhou Affiliated Hospital of Fujian Medical University Zhangzhou China; ^2^ Department of Hematology Fujian Institute of Hematology Fujian Provincial Key Laboratory of Hematology Fujian Medical University Union Hospital Fuzhou China; ^3^ Minxi Vocational & Technical College Longyan China; ^4^ Third Institute of Oceanography Ministry of Natural Resources Xiamen China

**Keywords:** DNA methylation, IRAK1, miR‐204, NF‐κB, T cell acute lymphoblastic leukaemia

## Abstract

T cell acute lymphoblastic leukaemia (T‐ALL) is a highly aggressive haematological cancer of the bone marrow. The abnormal expression of microRNAs (miRNAs) is reportedly involved in T‐ALL development and progression. Thus, we aimed to decipher the involvement of miR‐204 silencing mediated by DNA methylation in the occurrence of T cell acute lymphoblastic leukaemia (T‐ALL). miR‐204 expression was determined in bone marrow and peripheral blood samples from T‐ALL patients by real‐time quantitative PCR (RT‐qPCR) with its effect on cell proliferation evaluated by functional assays. In addition, bisulphite sequencing PCR was employed to detect the DNA methylation level of the miR‐204 promoter region, and the binding site between miR‐204 and IRAK1 was detected by luciferase assay. We found that miR‐204 was down‐regulated in T cells of T‐ALL patients, which was caused by the increased DNA methylation in the promoter region of miR‐204. Moreover, overexpression of miR‐204 inhibited T‐ALL cell proliferation while enhancing their apoptosis through interleukin receptor‐associated kinase 1 (IRAK1), which enhanced the expression of matrix metalloproteinase‐2 (MMP‐2) and MMP‐9 through activation of p‐p65. Thus, miR‐204 modulated MMP‐2 and MMP‐9 through IRAK1/NF‐κB signalling pathway, which was confirmed by in vivo assay. Taken together, DNA methylation‐mediated miR‐204 silencing increased the transcription of IRAK1, thus activating the NF‐κB signalling pathway and up‐regulating the downstream targets MMP‐2/MMP‐9.

## 1 INTRODUCTION

T cell acute lymphoblastic leukaemia (T‐ALL) is a highly aggressive haematological malignancy caused by the overproduction of T cells. T‐ALL contributes to 15% of ALL cases in children and 25% of those in adults, and its overall survival rate is quite low, being only 60%‐70% in children and 30%‐40% in adults.^1^, ^2^, ^3^ Although recent papers have reported the potential molecular mechanisms leading to T‐ALL,^4^, ^5^ sufficient understanding of the metastatic mechanisms of T‐ALL is still lacking and in need for further investigation.

Increasing evidence suggests that methylation of microRNAs (miRNAs) and activation of the nuclear factor kappa‐light‐chain‐enhancer of activated B cells (NF‐κB) signalling pathway are involved in T‐ALL development and progression.^6^ Indeed, NF‐ĸB signalling is an important pathway in T‐ALL cells, whereby sirtuin1 can physically interact with and deacetylate the NF‐ĸB subunit of p65/RELA at lysine 310 residue, thus resulting in transcriptional inhibition.^7^ Moreover, NF‐κB which is recognized as an important factor in normal inflammatory, immune and carcinogenesis, is found to be activated in T‐ALL.^8^ Importantly, activation of NF‐κB in cancer‐associated leucocytes drives cytokine secretion that facilitates metastasis *via* activation of the C‐X‐C chemokine receptor type 7 (CXCR7).^9^ Yang et al revealed that miR‐101 reduced T‐ALL cell proliferation and invasion by targeting CXCR7/signal transducer and the activator of transcription 3 (STAT3) signalling pathway.^10^

Previous research has indicated that miR‐204 expression is down‐regulated in T‐ALL,^11^ and that the cancer development and progression is promoted by miR‐204 silencing due to DNA methylation of the miR‐204 promoter.^12^ Interleukin‐1 receptor‐associated kinase (IRAK) contributes significantly in the pathogenesis of inflammatory autoimmune disorders,^13^ and T‐ALL cells express elevated levels of IRAK1 mRNA, as well as increased proportions of activated IRAK1.^14^ Bioinformatics tools predicted that IRAK1 was a possible target of miR‐204. It has previously been reported that IRAK1 was an upstream signalling component of the NF‐κB signalling pathway.^15^ Of note, previous research has shown that NF‐κB was overexpressed in T‐ALL cells, indicating that NF‐κB signalling pathway may be potentially involved in the molecular mechanism of T‐ALL.^16^ However, the specific mechanisms underlying DNA methylation of miR‐204 promoter in T‐ALL remain to be identified.

In this study, we measured the expression of miR‐204 and the methylation level of its promoter region in specimens from T‐ALL patients and in T‐ALL cell lines, and studied the effects of the downstream regulatory gene IRAK1 in changing the expression of NF‐κB and ultimately promoting proliferation and metastasis of T‐ALL cells. In so doing, we test the hypothesis that miR‐204 silencing mediated by DNA methylation regulates the IRAK1/NF‐κB signalling in the development of T‐ALL.

## 2 MATERIALS AND METHODS

### 2.1 Ethics statement

This study has been reviewed and approved by the Medical Ethics Committee of Zhangzhou Affiliated Hospital of Fujian Medical University, and all patients signed informed consent. The experiments involved animals were performed with approval from the institutional Animal Care and Use Committee of Zhangzhou Affiliated Hospital of Fujian Medical University.

### 2.2 Clinical tissue samples

Peripheral blood and bone marrow samples were collected from 16 healthy volunteers (the normal group) and 32 patients who were diagnosed as T‐ALL (the T‐ALL group) at Zhangzhou Affiliated Hospital of Fujian Medical University from 2012 to 2016. The patients had received no clinical treatment prior to sampling. The CD3^+^ kit (Invitrogen, Carlsbad, CA, USA) and CD2^+^ kit (STEMCELL Tech., Vancouver, BC, Canada) were used to sort the T cells from blood of healthy volunteers and T‐ALL patients.

### 2.3 T‐ALL mouse model

Female NSG mice (4‐6 weeks old) purchased from Weitonglihua Experimental Animal Corporation (Beijing, China) were randomized into three groups (n = 12/group). The mice were bred in a specific pathogen‐free (SPF) rodent feeding room and kept at 40%‐60% humidity and 22 ± 1°C with a 12 hours‐light/dark cycle. Jurkat cells transfected with mimic‐NC + overexpression‐negative control (oe‐NC), mimic‐miR‐204 + oe‐NC or mimic‐miR‐204 + oe‐p65 were injected into mice by tail‐vein injection, to establish the T‐ALL mouse model. T‐ALL cells collected from patients were injected into another group of mice. On day 7, miR‐204 mimic was injected into the mice, which hosted similar numbers of T‐ALL cells as revealed by flow cytometry. On day 21, the mice were sacrificed by anaesthesia overdose and the number of T‐ALL cells (CD7^+^) in the bone marrow and peripheral blood leucocytes were counted using flow cytometry.

### 2.4 Cell culture and transfection

The HEK293T cell line and the human T‐ALL cell line (Jurkat) were purchased from ATCC (Manassas, VA, USA). Jurkat cells were grown in the RPMI1640 (Gibco, Thermo Fisher Scientific, Waltham, MA, USA) supplemented with 10% foetal bovine serum (FBS, Gibco) and 1% penicillin/streptomycin, while HEK293T in the 10% FBS and 1% penicillin/streptomycin‐contained DMEM (HyClone, Logan, UT, USA). All cells were cultured at 37°C in a 5% CO_2_ humidified atmosphere. When the cell density reached 80%, the cells were transfected as per the protocols of the lipofectamin 2000 kit (11668‐019, Invitrogen, New York, California, USA). After 48 hours transfection, the cells were then cultured in the 10% FBS‐contained medium for 24‐48 hours. miR‐204 mimic and miR‐204 inhibitor were purchased from GenePharma (Shanghai, China).

HEK293T cells were transfected with (1) mimic‐NC + WT‐IRAK1, (2) mimic‐miR‐204 + WT‐IRAK1, (3) mimic‐NC + Mut‐IRAK1 and (4) mimic‐miR‐204 + Mut‐IRAK1; Jurkat cells were transfected with (1) mimic‐NC, (2) mimic‐miR‐204, (3) inhibitor‐NC, (4) inhibitor‐miR‐204, (5) mimic‐NC + oe‐NC, (6) mimic‐miR‐204 + oe‐NC, (7) mimic‐miR‐204 + oe‐IRAK1, (8) mimic‐NC + oe‐NC, (9) mimic‐miR‐204 + oe‐NC, (10) mimic‐miR‐204 + oe‐p65, (11) oe‐NC + si‐NC, (12) oe‐IRAK1 + si‐NC or (13) oe‐IRAK1 + si‐p65.

### 2.5 CCK‐8 assay and Hoechst 33342 and Propidium iodide (PI) double staining

Cells were seeded in 96‐well plates at 20 000 cells/well. After transfection for 5 days in the incubator, 100 μL portions of cell counting kit‐8 solution (CCK‐8, Beyotime, Shanghai, China) was added to each well and incubated at 37°C in the dark for 1 hour. Then, a microplate reader (Bio‐Rad, Hercules, CA, USA) was used to measure the absorbance at the wavelength of 560 nm. For Hoechst 33342 and PI double staining assays, cells were incubated with Hoechst 33342 (5 μg/mL) and PI (10 μg/mL) at room temperature for 10 minutes. Fluorescence was observed using a fluorescence microscope.

### 2.6 Apoptosis assay

Cell apoptosis in vitro was determined by an Annexin V‐FITC/PI double staining assay. Annexin V‐FITC apoptosis detection kit (BD Pharmingen, Franklin Lakes, NJ, USA) was used to detect the apoptosis of cells, which were counted after staining by flow cytometry using the FACSCalibur flow cytometer (BD Biosciences, San Jose, CA, USA).

### 2.7 RNA extraction and real‐time quantitative PCR (RT‐qPCR)

Total RNA was extracted using the Trizol kit (15596026, Invitrogen, Carlsbad, CA, USA). Synthesis of cDNA from mRNA was generated using the PrimeScript RT reagent kit (RR047A, Takara, Japan), following the instructions provided by the manufacturer. Synthesis of cDNA of miRNA was carried out using the miRcute miRNA First‐strand cDNA synthesis kit (Tiangen Biotech., Beijing, China). cDNA was subjected to RT‐qPCR using Fast SYBR Green PCR kit (Applied Biosystems, Foster City, CA, USA) with the ABI 7300 RT‐PCR instrument (Applied Biosystems), with each reaction run in triplicate. Glyceraldehyde‐3‐phosphate dehydrogenase (GAPDH) served as the internal reference protein. Individual miRNA‐specific forward primer and mRNA primer information are listed in Table 1. Results were calculated using the 2^−△△CT^ method.

**Table 1:** RT‐qPCR Primer sequences

Gene	Sequence
IRAK1	Forward 5′‐TCAGAACGGCTTCTACTGCCTG‐3′
	Reverse 5′‐TACCCAGAAGGATGTCCAGTCG‐3′
GAPDH	Forward 5′‐GTCTCCTCTGACTTCAACAGCG‐3′
	Reverse 5′‐ACCACCCTGTTGCTGTAGCCAA‐3′
miR‐204	Forward 5′‐GGGCTTCCCTTTGTCATCCTAT‐3′
	Reverse 5′‐CCAGTGCAGGGTCCGAGGT‐3′
U6	Forward 5′‐GACACGCAAATTCGTGAAGCG‐3′
	Reverse 5′‐TCCAGTGCAGGGTCCGAG‐3′

### 2.8 Protein extraction and quantification

Proteins were extracted using protease inhibitor‐contained RIPA buffer (Boster, Wuhan, China). The concentration of total proteins was quantified using a bicinchoninic acid (BCA) protein assay kit (Boster, Wuhan). The protein sample was separated using freshly prepared 10% sodium dodecyl sulphate polyacrylamide gel electrophoresis (SDS‐PAGE) and then electrotransferred onto polyvinylidene fluoride membranes. Non‐specific binding was blocked with 5% bull serum albumin for 2 hours, and primary antibodies (Cell Signaling Technologies, Danvers, MA, USA) were added. Membranes were incubated with HRP‐labelled secondary antibodies for 1 hour at room temperature, subsequently incubated with the enhanced chemiluminescence (EMD Millipore, Chicago, IL, USA) for 1 minutes and autoradiographed using X‐ray film. The grey value of target protein bands was quantified using Image J software, with β‐actin used for normalization. Primary antibodies used in this study: anti‐IRAK1 antibody (CST4359), anti‐β‐actin antibody (CST4970), anti‐p‐p65 antibody (CST3033), anti‐p65 antibody (CST8242), anti‐MMP‐2 antibody (CST40994) and anti‐MMP‐9 antibody (CST8242).

### 2.9 Dual‐luciferase reporter assay

The binding site between miR‐204 and IRAK1 was predicted by the Primer Premier 5.0 software, and the binding sequences were obtained to construct luciferase‐tagged plasmids. The fragments of IRAK1 3’ untranslated regions (3’UTR) containing the assumed wild‐type (WT) miR‐204 binding sites (IRAK1‐WT) or mutant (MUT) binding sites (IRAK1‐MUT) were, respectively, inserted into the 3’UTR of the luciferase reporter gene to construct recombinant PGLO vectors. The desired luciferase reporter plasmids, IRAK1‐WT or IRAK1‐MUT, were delivered into Jurkat cells together with miR‐204 mimic or NC mimic (GenePharma, Shanghai, China). After incubation for 24 hours, the cells were lysed and centrifuged at 26 000 *g* for 1 minutes. The luminescence of firefly luciferase in the supernatant was determined using a dual‐luciferase reporter assay system kit (E1910, Promega, Madison, WI, USA) as per the instructions provided by the manufacturer.

### 2.10 Bisulphite DNA sequencing analysis

Purified DNA was treated with sodium bisulphite according to the EZ DNA Methylation‐Gold Kit protocol (Zymo Research, Milan, Italy) followed by PCR reaction. The PCR product was purified by 2% agarose electrophoresis and sequenced by PyroMark MD Q96 MD System (Qiagen, Hilden, Germany) with a sequencing primer. Percent methylation for each CpG cytosine was determined using Pyro Q‐CpG Software (Qiagen, Hilden, Germany). Upstream primers: 5′‐[Btn]TGGTTTTTTTTTAATTAAGTTAGTAAAGT‐3′; Downstream primer: 5′‐ACAACCTACACAAAACAACCTATAATC‐3′.

### 2.11 Statistical analysis

All statistical analyses were completed with SPSS 21.0 software (IBM, Armonk, NY, USA). Data were shown as the mean ± standard deviation from at least three independent experiments performed in triplicate. First, tests for normality and homogeneity of variance were performed to confirm that the data conformed to normality and homogeneity of variance. The data between two different groups were analysed by unpaired *t* test, the data between multiple groups were analysed by one‐way analysis of variance (ANOVA) and Tukey's *post hoc* test, and the data between multiple groups at different time points were analysed by two‐way ANOVA. A value of *P* < 0.05 was indicative of significant statistical difference.

## 3 RESULTS

### 3.1 DNA methylation induces decreased miR‐204 in T‐ALL

**FIGURE 1:**
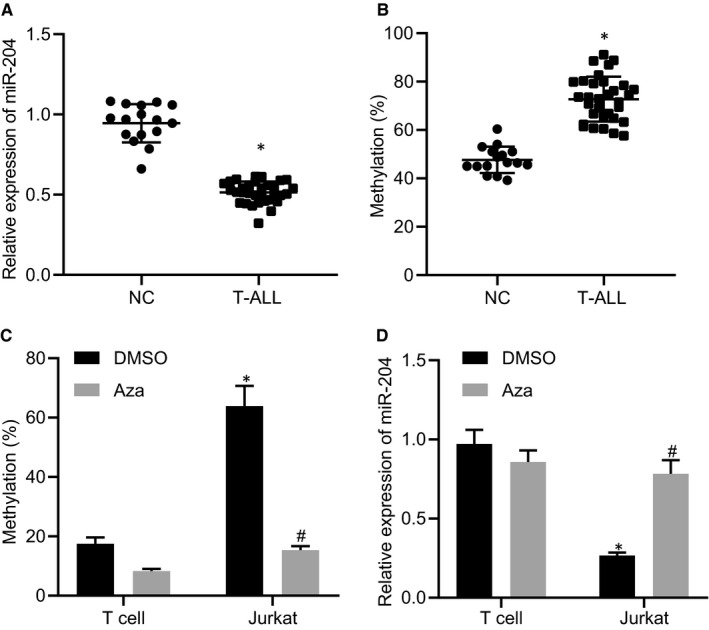
DNA methylation inhibits the expression of miR‐204. A, The expression level of miR‐204 in T cells collected from T‐ALL patients and healthy volunteers, normal = 16, T‐ALL = 32. B, Methylation of the promoter region of miR‐204 in T cells collected from T‐ALL patients and healthy volunteers, normal = 16, T‐ALL = 32. C, Methylation levels of the promoter region of miR‐204 in normal T cell and Jurkat cell line, before and after demethylation. D, The expression levels of miR‐204 in normal T cell and Jurkat cell line, before and after demethylation. In panels A and B, *indicates*P* < 0.05 compared with healthy volunteers. In panel C and D, *indicates *P* < 0.05 compared with DMSO, #indicates *P* < 0.05 compared with Jurkat cell line, respectively. Data are shown as the mean ± standard deviation from at least three independent experiments performed in triplicate. Comparisons of means between two groups were carried out using a *t* test and multiple comparisons were performed by two‐way ANOVA

Previous studies suggested that miR‐204 under‐expressed in the T cells of T‐ALL patients.^11^ To determine the role of miR‐204 in T‐ALL, here we first analysed the expression of miR‐204 in T cells collected from 32T‐ALL patients and 16 healthy volunteers using RT‐qPCR. The results showed that miR‐204 expression was down‐regulated in T cells of T‐ALL patients compared to those of healthy volunteers (Figure 1A), which is consistent with the previous report that DNA methylation could lead to transcriptional silencing of miR‐204 in squamous cell carcinoma of skin.^12^ Bisulphite DNA sequencing subsequently showed that the DNA methylation level of the miR‐204 promoter region CpG (−26, −28 and −40) of the T‐ALL group were significantly higher than that of the normal group (Figure 1B). Besides, we found that higher DNA methylation levels and lower miR‐204 expression levels in the Jurkat cell line, while demethylation by treatment with 5‐aza‐2’‐deoxycytidine (Aza) reversed these changes (Figure 1C,D). As we expected, DNA methylation in the promoter region directly down‐regulated the miR‐204 expression when T‐ALL developed.

### 3.2 Overexpression of miR‐204 inhibits the proliferation and enhances apoptosis of T‐ALL cells

**FIGURE 2:**
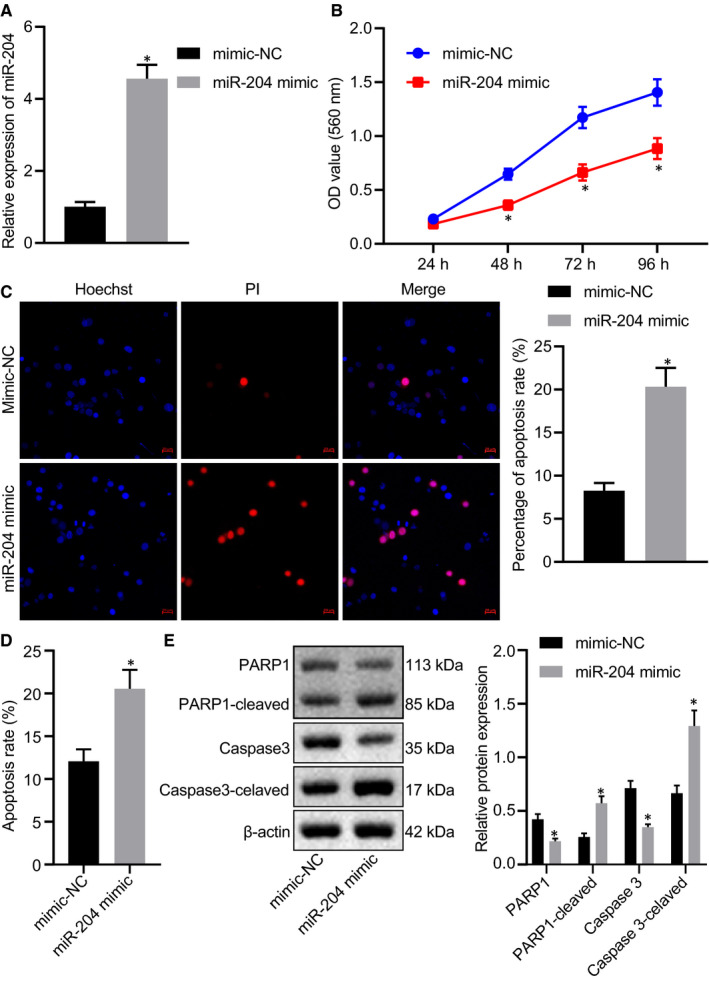
Overexpression of miR‐204 inhibits the proliferation and enhances apoptosis of T‐ALL cells. A, RT‐qPCR determination of miR‐204 expression levels in miR‐204 mimic‐transfected Jurkat cells. B, Cell proliferation in miR‐204 mimic‐transfected Jurkat cells assessed by CCK‐8 assay. C, The effect of miR‐204 on T‐ALL cell viability observed by Hoechst and PI staining (20 µm). D, T‐ALL cell apoptosis assessed by Annexin V/PI staining. E, Western blot analysis of the expression of PARP1, PARP1‐cleaved, Caspase3 and Caspase3‐cleaved. *indicates *P* < 0.05 compared with mimic NC. Data are shown as the mean ± standard deviation from at least three independent experiments performed in triplicate. Comparisons of data were carried out using *t* test, and comparisons of data at different time points were performed by two‐way ANOVA

To unveil the effect of miR‐204 on the proliferation of T‐ALL cells, we transfected miR‐204 into the Jurkat cells. RT‐qPCR confirmed that miR‐204 expression was significantly increased in miR‐204 mimic‐transfected Jurkat cells (Figure 2A). By using CCK‐8 assay as well as Hoechst and PI double staining assay, we found that the proliferation of T‐ALL cells was inhibited (Figure 2B) and cell death was increased (Figure 2C) upon overexpression of miR‐204 in Jurkat cells. To further determine the apoptosis rate of T‐ALL cells, we employed an in vitro Annexin V‐FITC/PI double staining assay to sort the cells by flow cytometry. We found increased numbers of Annexin V‐positive cells after overexpression of miR‐204 (Figure 2D). Similarly, the results of Western blot analysis depicted that the expression of PARP‐1‐cleaved and Caspase3‐cleaved was increased (Figure 2E), which demonstrated that the overexpression of miR‐204 enhanced T‐ALL cell apoptosis.

### 3.3 miR‐204 directly targets the 3’UTR of IRAK1 in Jurkat cells

**FIGURE 3:**
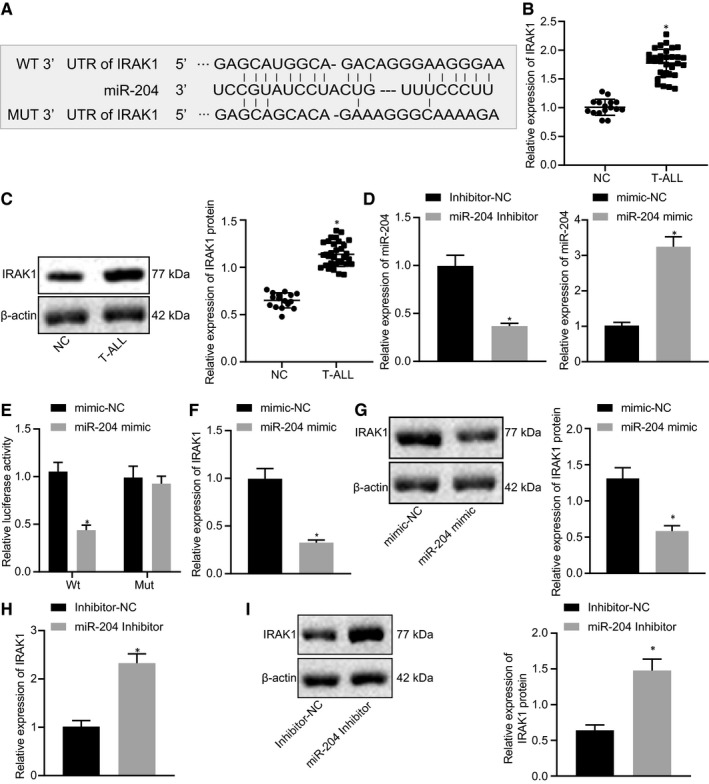
miR‐204 targets IRAK1. A, The predicted binding sites of miR‐204 on 3’ UTR of IRAK1 and the mutant IRAK1 3’ UTR sequence at the binding site. B and C, RT‐qPCR and Western blot analysis of expression of IRAK in T‐ALL patients’ T cells, normal = 16, T‐ALL = 32. D, RT‐qPCR determination of miR‐204 expression in miR‐204 mimic or miR‐204 inhibitor‐treated Jurkat cells. E, The regulatory effect of miR‐204 on IRAK1 3’UTR assessed by luciferase assay. F and G, RT‐qPCR and Western blot analysis of IRAK1 expression in miR‐204 mimic‐transfected Jurkat cells. H and I, RT‐qPCR and Western blot analysis of IRAK1 in miR‐204 inhibitor‐transfected Jurkat cells. *indicates *P* < 0.05 compared with NC, mimic‐NC or inhibitor‐NC. Data are shown as the mean ± standard deviation from at least three independent experiments performed in triplicate. Comparisons of means between two groups were carried out using a *t* test

To study the effect of miR‐204 on T‐ALL, we first predicted IRAK1 as a target of miR‐204 through scrutiny of the relevant website (Figure 3A). To further confirm the role of IRAK1 in the occurrence of T‐ALL, we detected the levels of IRAK1 mRNA and protein. Our results from RT‐qPCR and Western blot analysis demonstrated a significant increase of IRAK mRNA and protein expression in T cells of the T‐ALL group compared to the normal group (Figure 3B,C). Additionally, RT‐qPCR determination results also demonstrated that miR‐204 expression levels were significantly decreased/increased in miR‐204 inhibitor/mimic‐transfected Jurkat cells, respectively (Figure 3D). Subsequently, dual‐luciferase reporter assays displayed that the overexpression of miR‐204 in HEK293T cells significantly suppressed the luciferase activities of IRAK1‐3’ UTR WT, but exerted no such effect on the luciferase activities of IRAK1‐3’ UTR MUT (Figure 3E). We then performed RT‐qPCR and Western blot analysis to determine whether miR‐204 regulated IRAK1 expression, which revealed the overexpression of miR‐204 down‐regulated IRAK1 mRNA and protein expression levels in Jurkat cells (Figure 3F,G). Conversely, Jurkat cells transfected with miR‐204 inhibitor showed up‐regulated levels both of IRAK1 mRNA and protein (Figure 3H,I). As we had expected, miR‐204 negatively modulates IRAK1 expression levels in Jurkat cells at the post‐transcriptional level.

### 3.4 Inhibition of IRAK1 modulated by miR‐204 inhibits the proliferation of T‐ALL cells and enhances apoptosis

**FIGURE 4:**
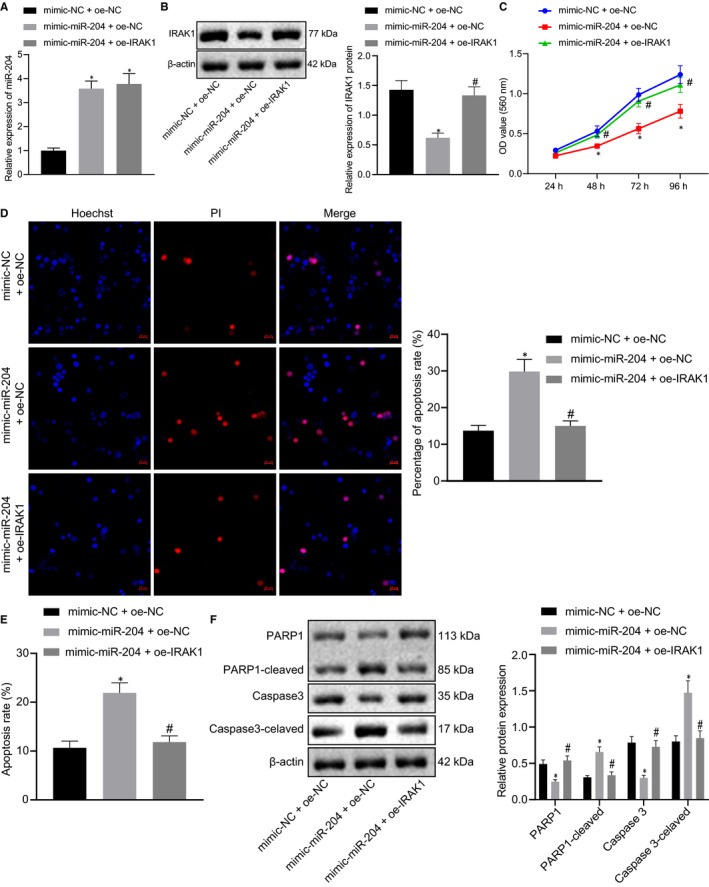
Inhibition of IRAK1 modulated by miR‐204 inhibits the proliferation of T‐ALL cells and enhances apoptosis. A, Expression levels of miR‐204 in Jurkat cells determined by RT‐qPCR. B, Expression levels of IRAK1 protein in Jurkat cells examined by Western blot analysis. C, Cell proliferation detected by CCK‐8 assay. D, Cell viability assessed Hoechst and PI staining (20 µm). E, Cell apoptosis assessed by Annexin V/PI. F, Western blot analysis of PARP1, PARP‐1 cleaved, Caspase 3 and Caspase 3‐cleaved expression. *indicates *P* < 0.05 compared with the mimic‐NC + oe‐NC‐treated group. #indicates *P* < 0.05 compared with mimic‐miR‐204 + oe‐NC‐treated group. Data are shown as the mean ± standard deviation from at least three independent experiments performed in triplicate. Comparisons of means between multiple groups were carried out using one‐way ANOVA with Tukey's *post hoc* analysis. Multiple comparisons with different time points were performed by two‐way ANOVA

To further unveil whether miR‐204 affects T‐ALL through modulation of IRAK1, we transfected Jurkat cells with mimic‐NC + oe‐NC, mimic‐miR‐204 + oe‐NC and mimic‐miR‐204 + oe‐IRAK1. RT‐qPCR results showed that miR‐204 expression in the mimic‐miR‐204 + oe‐NC and mimic‐miR‐204 + oe‐IRAK1 groups were significantly increased compared to the mimic‐NC + oe‐NC group (Figure 4A). Compared with the mimic‐NC + oe‐NC group, protein expression of IRAK1 was significantly decreased in Jurkat cells transfected with mimic‐miR‐204 + oe‐NC, while the expression levels showed no significant difference in mimic‐miR‐204 + oe‐IRAK1‐transfected Jurkat cells (Figure 4B). CCK‐8 assay revealed that transfection of miR‐204 mimic inhibited the proliferation of the Jurkat cells, but that the addition of overexpressed IRAK1 restored their growth rate (Figure 4C). Likewise, as shown in Figure 4D, overexpression of miR‐204 increased cell death, while the co‐treatment of mimic‐miR‐204 and oe‐IRAK1 had no effect on cell death. To demonstrate further the effects of different treatment methods on the apoptosis of Jurkat cells, we used flow cytometry to sort the cells stained with Annexin V/PI. Compared with transfection of mimic‐NC + oe‐NC treatment, overexpression of miR‐204 and NC increased the number of Annexin V‐positive cells, while their number was unaffected by overexpression of miR‐204 and IRAK1 were both overexpressed (Figure 4E). As evidenced by Western blot analysis, the PARP‐1‐cleaved and Caspase3‐cleaved were both increased in mimic‐miR‐204 + oe‐NC‐treated Jurkat cells, and the overexpression of miR‐204 and IRAK1 reduced apoptosis, whereas no such significant effects were seen in the mimic‐NC + oe‐NC group (Figure 4F). The above results supported the prediction that miR‐204 impeded the proliferation of T‐ALL cells and promotes their apoptosis through inhibiting IRAK1.

### 3.5 MiR‐204 inhibits IRAK1 to enhance the expression of MMP‐2/MMP‐9 through activation of p‐p65

**FIGURE 5:**
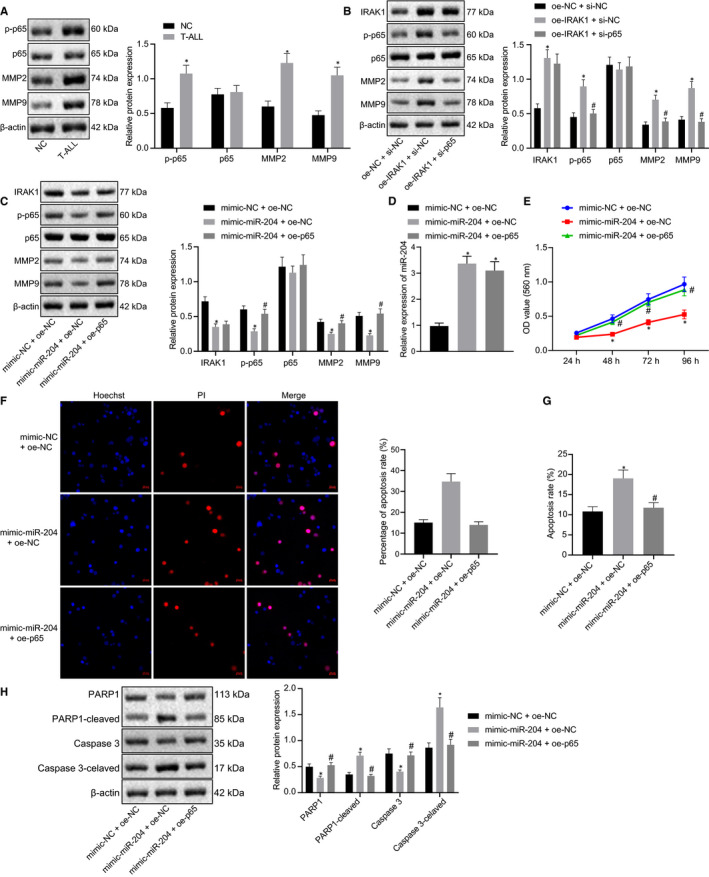
IRAK1 enhances the expression of MMP‐2/MMP‐9 through the activation of p‐p65. A, Expression levels of p‐p65, p65, MMP2 and MMP9 in T cells determined by Western blot, normal = 16, T‐ALL = 32. B, Expression levels of IRAK1, p‐p65, MMP2 and MMP9 in oe‐IRAK1 + si‐NC‐treated cells determined by Western blot analysis. C, Expression levels of IRAK1, p‐p65, MMP2 and MMP9 in mimic‐miR‐204‐treated cells determined by Western blot analysis. D, Expression levels of miR‐204 in the mimic‐miR‐204 + oe‐NC group and the mimic‐miR‐204 + oe‐p65 group determined by RT‐qPCR. E, Cell proliferation determined by CCK‐8 assay. F, Cell viability assessed by Hoechst and PI staining (20 µm). G, Cell apoptosis assessed by Annexin V/PI. H, Western blot analysis of PARP1, PARP‐1 cleaved, Caspase 3 and Caspase 3‐cleaved expression. *indicates *P* < 0.05 compared with mimic‐NC + oe‐NC group, #indicates *P* < 0.05 compared with mimic‐miR‐204 + oe‐NC group. Data are shown as the mean ± standard deviation from at least three independent experiments performed in triplicate. Comparisons of means between multiple groups were carried out using one‐way ANOVA with Tukey's *post hoc* analysis. Multiple comparisons with different time points were performed by two‐way ANOVA

To confirm the role of MMP‐2 and MMP‐9 in the occurrence of T‐ALL, we investigated the effect of related proteins on T‐ALL cells using Western blot analysis. It has been known for some time that in NK cells IRAK1 is the upstream effector of the NF‐κB signalling pathway,^15^ which is highlyexpressed in T‐ALL patients in proportion to disease progression.^16^ MMP‐2 and MMP‐9 are important downstream proteins of the NF‐κB signalling pathway,^17^ and play vital roles in T‐ALL.^18^ Our results proved that the expression levels of p‐p65, MMP‐2 and MMP‐9 in T cells from T‐ALL patients were significantly increased compared with the levels in healthy volunteers (Figure 5A), suggesting that MMP‐2 and MMP‐9 contributed to the occurrence of T‐ALL.

Subsequently, oe‐NC + si‐NC, oe‐IRAK1 + si‐NC and oe‐IRAK1 + si‐p65 were transfected into Jurkat cells to determine whether IRAK1 affects the expression of MMP‐2 and MMP‐9 through the NF‐κB signalling pathway. As shown in Figure 5B, expression levels of IRAK1, p‐p65, MMP2 and MMP9 in oe‐IRAK1 + si‐NC‐treated cells were higher than in the oe‐NC + si‐NC‐treated cells. As expected, the expression levels of p‐p65, MMP2 and MMP9 were decreased when p‐65 was knocked down, which demonstrated that IRAK1 up‐regulated the expression of MMP2 and MMP9 by activating the NF‐κB signalling pathway. Likewise, mimic‐NC + oe‐NC, mimic‐miR‐204 + oe‐NC and mimic‐miR‐204 + oe‐p65‐treated Jurkat cells were used to investigate the effect of miR‐204 on NF‐κB signalling pathway and the regulation of MMP‐2 and MMP‐9. Western blot results showed that the overexpression of miR‐204 significantly down‐regulated IRAK1, p‐p65, MMP2 and MMP9 expression, while the expression levels of p‐p65, MMP2 and MMP9 were increased when p65 was up‐regulated (Figure 5C). To further demonstrate the role of miR‐204, we conducted RT‐qPCR to analyse the expression levels in different transfected Jurkat cells, which showed that miR‐204 was increased both in the mimic‐miR‐204 + oe‐NC group and the mimic‐miR‐204 + oe‐p65 group, compared with the mimic‐NC + oe‐NC group (Figure 5D). As we had expected, miR‐204 diminishes the expression of MMP2 and MMP9 through down‐regulating the expression of p‐p65.

Furthermore, we performed the CCK‐8 assay to demonstrate that overexpression of miR‐204 inhibited the proliferation of the Jurkat cells, whereas overexpression of miR‐204 and p‐65 restored the proliferation (Figure 5E). As shown in Figure 5F, overexpression of miR‐204 increased cell death, while in the cells transfected with mimic‐miR‐204 + oe‐p65, the death was reduced to no significant difference from that of the mimic‐NC + oe‐NC group. To further demonstrate the effect of different treatments on the apoptosis of Jurkat cells, we used flow cytometry to sort the cells stained with Annexin V/PI. Compared with transfection of mimic‐NC + oe‐NC, overexpression of miR‐204 and NC increased the number of Annexin V positive cells, while their number was unaffected when miR‐204 and p65 were both overexpressed (Figure 5G). As evidenced by Western blot, the cleaved PARP‐1 and Caspase3 levels were both increased in the mimic‐miR‐204 + oe‐NC‐treated Jurkat cells, while the overexpression of miR‐204 and p65 restored their levels (Figure 5H). The aforementioned results show that miR‐204 inhibits T‐ALL cells proliferation and enhances apoptosis through inhibition of p65.

### 3.6 miR‐204 modulates MMP‐2 and MMP‐9 expression through the IRAK1/NF‐κB signalling pathway in vivo

**FIGURE 6:**
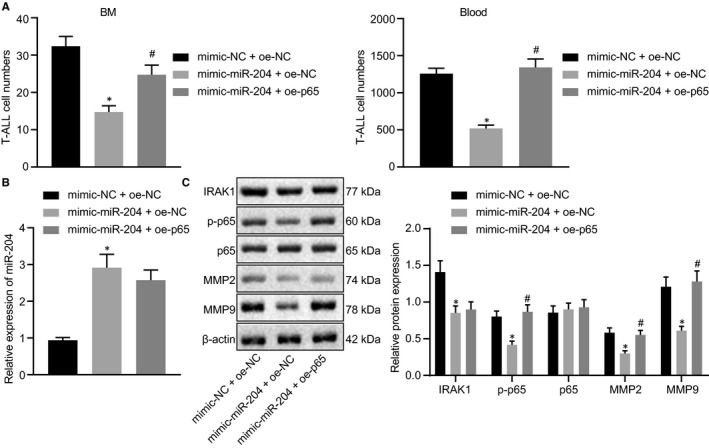
miR‐204 modulates MMP‐2 and MMP‐9 through the IRAK1/NF‐κB signalling pathway in vivo. A, The number of T‐ALL cells in the bone marrow and peripheral blood collected from mice injected with mimic‐NC + oe‐NC, mimic‐miR‐204 + oe‐NC or mimic‐miR‐204 + oe‐p65‐transfected Jurkat cells, on day 21, n = 12. B, Expression levels of miR‐204 in Jurkat cells transfected with mimic‐miR‐204 + oe‐NC and mimic‐miR‐204 + oe‐p65 determined by RT‐qPCR. C, Expression levels of IRAK1, p‐p65, MMP2, and MMP9 in T cells from mimic‐miR‐204 + oe‐NC‐treated mice assessed by Western blot analysis. *indicates *P* < 0.05 compared with the mimic‐NC + oe‐NC group. #indicates *P* < 0.05 compared with mimic‐miR‐204 + oe‐NC group. Data are shown as the mean ± standard deviation from at least three independent experiments performed in triplicate. Comparisons of means between multiple groups were carried out using one‐way ANOVA with Turkey's *post hoc* test. Multiple comparisons with different time points were performed by two‐way ANOVA. N = 8

To determine ifmiR‐204 affects the occurrence of T‐ALL disease in mice by regulating the NF‐κB signalling pathway, we administered mimic‐NC + oe‐NC, mimic‐miR‐204 + oe‐NC or mimic‐miR‐204 + oe‐p65‐transfected Jurkat cells via the tail vein of groups of mice. The expression levels of miR‐204 in T cells from mouse bone marrow and peripheral blood, as well as the number of T‐ALL cells, were detected by flow cytometry on day 21. The results showed that the number of T‐ALL cells in the mimic‐miR‐204 + oe‐NC group was significantly reduced compared to the mimic‐NC + oe‐NC group (Figure 6A), showing that overexpression of miR‐204 inhibits the occurrence of T‐ALL in vivo. Compared with the mimic‐miR‐204 + oe‐NC group, the mice treated with cells transfected with mimic‐miR‐204 + oe‐p65 showed increased numbers of T‐ALL cells (Figure 6A), which proves that regulation of T‐ALL by miR‐204 depends on the NF‐κB signalling pathway. Subsequently, RT‐qPCR and Western Blot were used for further verification. The results showed increased miR‐204 expression in Jurkat cells transfected with mimic‐miR‐204 + oe‐NC (Figure 6B), but decreased expression of IRAK1, p‐p65, MMP2 and MMP9 compared to the mimic‐NC + oe‐NC group (Figure 6C). Compared with the mimic‐miR‐204 + oe‐NC group, co‐overexpression of miR‐204 and p65 up‐regulated the p‐p65 in T cells, and the expression levels of MMP‐2 and MMP‐9 were also increased (Figure 6C). Therefore, we conclude miR‐204 regulates the expression of MMP‐2 and MMP‐9 through the IRAK1/NF‐κB signalling pathway, thereby alleviating T‐ALL in vivo.

## 4 DISCUSSION

Recent papers have reported that methylation of miRNAs and activation of NF‐κB signalling pathway were involved in T‐ALL development and progression.^8^, ^19^ In our investigation, we explored the expression and functional role of miR‐204 in T‐ALL. The experimental data demonstrated that overexpression of miR‐204 up‐regulated the expression of MMP‐2 and MMP‐9 through IRAK1/NF‐κB signalling pathway, thereby alleviating T‐ALL.

DNA methylation of miRNAs has become a commonly studied epigenetic regulatory mechanism for modifying gene expression.^20^ Our investigation detected miR‐204 expression and promoter methylation levels in T‐ALL patients and T‐ALL cell lines, which led us to conclude that DNA methylation indeed reduced expression of miR‐204 in T‐ALL, whereas overexpression of miR‐204 could inhibit the proliferation of T‐ALL cells and promoted T‐ALL cell apoptosis. Zanette et al found that miRNAs could play a role in diverse biological processes including development, cell growth, apoptosis and haematopoiesis, and, more specifically, showed that miR‐204 was highly expressed in haematological malignancies.^21^ In addition, studies with reprogramming of miRNA networks in cancer and leukaemia suggested that hsa‐miR‐204 was deregulated and physically altered at the level of DNA copy number.^22^ Consistent with these findings, a prominent role of miR‐204 in T‐ALL has been reported whereby overexpression of miR‐204 could significantly suppress the migration and invasion ability of T‐ALL cells.^11^ Besides, Xia et al also reported that down‐regulated miR‐204 expression was related to several important pathways and mechanisms involved in tumorigenesis and progression.^23^

In our present study, we found that miR‐204 targeted inhibition of IRAK1 expression, thereby inhibiting T‐ALL cell proliferation and promoting T‐ALL cell apoptosis. IRAK1 is known as a widely expressed serine/threonine kinase, which has a regulatory effect on signalling downstream to Toll‐like and Interleukin‐1 Receptors, and furthermore, IRAK1 is reported to be highly expressed in all types of T‐ALL and to exert regulatory functions in T‐ALL cell lines.^24^ Partially consistent with our present findings, the expression of IRAK1 mRNA and the levels of IRAK1 have been found elevated in T‐ALL cells, and IRAK4 signalling was implicated in as having a critical role in T‐ALL proliferation and chemo‐resistance.^14^ Recent findings showed that that miR‐146a directly targets IRAK1 and TRAF6 to alleviate cardiac ischaemia and reperfusion injury.^25^ Hence, a wide body of evidence support our model that miR‐204 targets IRAK1 to suppress the proliferation and promote the apoptosis of T‐ALL cells.

Furthermore, IRAK1 enhances the expression of MMP‐2/MMP‐9 by activating downstream NF‐κB to promote the proliferation of T‐ALL cells. Mounting evidence indicates that MMPs facilitate the development of many cancers. MMP‐2 and MMP‐9 are assumed to be particularly important for cell transmigration, and the association of cell surface between MMP‐2, MMP‐9 and integrins has been implicated in the progression and growth of chronic myeloid leukaemia and acute myeloid leukaemia cells.^26^ In addition, the activity of MMP‐2 and MMP‐9 may be related to an aggravated course of acute myeloid leukaemia,^27^ implying an interplay between MMP‐2/MMP‐9 and T‐ALL. Importantly, NF‐κB is an essential regulator of immune function,^28^ and inhibition of the NF‐κB pathway can induce cell growth inhibition and apoptosis.^29^ Wu et al demonstrated that NF‐κB stimulated by Asb2α induced degradation and dissociation of IκBα, contributing to T‐ALL.^30^ Inhibition of NF‐κB signalling was shown to impair leukaemic T cell growth, which has also been observed in acute T cell leukaemia mouse models.^31^ Of note, others have documented that the activities of MMP‐2 and MMP‐9 were suppressed by kisspeptin through inhibition of the nuclear translocation of the NF‐κB.^32^ Similarly, Tu et al found that loss of miR‐146b‐5p up‐regulated MMP‐9 expression via the NF‐κB signalling pathway, to promote T‐ALL cell migration and invasion.^6^ Very importantly, previous evidence also highlighted that IRAK‐1 could lead to the activation of NF‐κB.^33^

**FIGURE 7:**
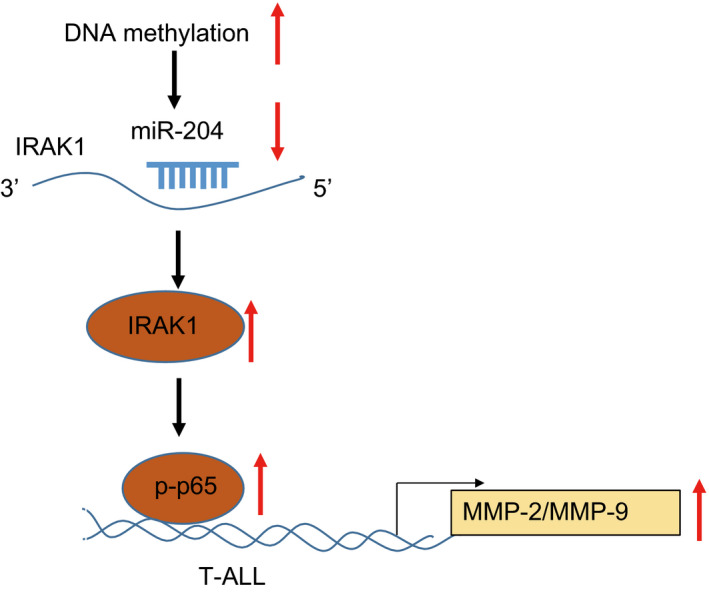
Overexpression of miR‐204 targets IRAK1, activating the downstream NF‐κB pathway and increasing MMP‐2/MMP‐9 expression in the downstream of NF‐κB, thereby inhibiting malignant phenotypes of T‐ALL cells. miR‐204 could be silenced by DNA methylation

In conclusion, our study elucidated the possible pathway that DNA methylation at the promotor region causes miR‐204 silencing, whereas overexpression of miR‐204 targets IRAK1, resulting in increased expression of the downstream NF‐κB signalling pathway and MMP‐2/MMP‐9, thereby alleviating T‐ALL (Figure 7). These findings suggest that miR‐204 impedes T‐ALL growth and metastasis by targeting IRAK1 and highlight the potential role of miR‐204/NF‐κB signal pathway in targeted therapy of T‐ALL patients.

## CONFLICT OF INTEREST

The authors declare that there is no conflict of interest.

## AUTHOR CONTRIBUTIONS

**Congmeng Lin:** Methodology (equal); Writing‐original draft (equal). **Dabing Chen:** Methodology (equal); Writing‐original draft (equal). **Tingting Xiao:** Methodology (equal); Writing‐original draft (equal). **Dandan Lin:** Methodology (equal); Writing‐original draft (equal). **Dayi Lin:** Data curation (equal); Formal analysis (equal). **Luhui Lin:** Data curation (equal); Formal analysis (equal). **Haojie Zhu:** Data curation (equal); Formal analysis (equal). **Jingjing Xu:** Resources (equal). **Wenwen Huang:** Resources (equal). **Ting Yang:** Resources (equal).

## Data Availability

The data that support the findings of this study are available from the corresponding author upon reasonable request.
